# Assessment of the Stability of Propellants Modified with Eco-Friendly Plasticizers

**DOI:** 10.3390/polym17223033

**Published:** 2025-11-15

**Authors:** Katarzyna Cieślak, Monika Izabella Wycech, Waldemar Tomaszewski

**Affiliations:** Department of High-Energetic Materials, Faculty of Chemistry, Warsaw University of Technology, Noakowskiego 3, 00-664 Warsaw, Poland

**Keywords:** nitrocellulose propellants, green plasticizers, citric acid esters, thermal stability, accelerated aging

## Abstract

The growing importance of sustainable technologies and environmental safety is promoting the implementation of green chemistry principles in the field of energetic materials. Traditionally, nitrocellulose-based propellants are plasticized with dibutyl phthalate (DBP), which is classified as a hazardous substance due to its toxicity and migration during storage. In this work, triethyl 2-acetylcitrate (ATEC) and tributyl 2-acetylcitrate (ATBC) were investigated as biodegradable and non-toxic alternatives to DBP. The objective of this study was to evaluate the thermal and chemical stability, physicochemical properties, and incorporation efficiency of these eco-friendly plasticizers in regard to propellants prepared from nitrocellulose of different origins and with nitrogen contents. The stability of the obtained propellants was assessed based on accelerated aging tests conducted in accordance with NATO STANAG 4582 and AOP-48 procedures. The results showed that both the ATEC- and ATBC-modified propellants meet the stability requirements corresponding to at least ten years of storage at 25 °C. The modified propellants showed slightly lower heats of combustion. Both plasticizers were effectively integrated into the nitrocellulose matrix without compromising density or stability. This study confirms that citric-acid-based plasticizers are promising green alternatives to conventional phthalates, offering improved environmental compatibility while maintaining the required performance and safety of nitrocellulose propellants.

## 1. Introduction

The rapid development of modern technologies and growing environmental awareness have promoted the adoption of green chemistry principles. Since their formulation in 1991 by Paul T. Anastas and John C. Warner, the twelve principles of green chemistry have served as a foundation for designing safer chemical processes and substances that minimize the use of toxic reagents and reduce the risk of fire or explosion [1]. In the field of energetic materials, especially in regard to the production of nitrocellulose-based propellants, there is increasing interest—driven by both regulatory and health considerations—in finding alternatives to conventional toxic components. 

According to the European Union’s REACH regulation (Registration, Evaluation, and Authorisation of Chemicals), substances classified as Substances of Very High Concern (SVHC), such as dibutyl phthalate (DBP), should be restricted or replaced with less hazardous alternatives [2]. DBP, commonly used as a plasticizer in nitrocellulose propellants, is toxic and exhibits a tendency to migrate from the polymer matrix during storage, leading to changes in ballistic properties and a reduction in chemical stability [3,4] Consequently, citric acid esters such as triethyl 2-acetylcitrate (ATEC) and tributyl 2-acetylcitrate (ATBC) are gaining an increasing amount of attention as non-toxic, biodegradable, and polymer-compatible plasticizers [5,6,7,8,9].

Recent studies have also explored the use of environmentally friendly stabilizers in polymer-based energetic systems. For example, Krutko et al. [10] demonstrated that organic antioxidants can effectively enhance thermo-oxidative resistance in pitch-based polymer composites. These findings support the relevance of searching for green stabilizers and plasticizers for propellant formulations, wherein inhibition of oxidative degradation is essential for long-term stability.

Recently, significant progress has also been made in the development of eco-friendly stabilizers for energetic formulations, including natural antioxidants, ionic stabilizers, and bio-based compounds capable of scavenging nitrocellulose degradation products [11]. Research suggests that these novel stabilizers and citrate-based plasticizers improve storage stability not only for propellants but also for explosive compositions and energetic polymer systems [12]. Of importance in this context is the work demonstrating environmentally compatible additives for NC-based energetic systems and their thermal stability behavior [13].

Stabilizers and plasticizers play complementary roles in propellant formulations. Stabilizers act by scavenging nitrogen oxides and reactive radicals formed during nitrocellulose decomposition, slowing autocatalytic reactions and extending storage lifetime [11,14]. Plasticizers reduce the glass-transition temperature and increase polymer chain mobility, enhancing elasticity and structural integrity [15,16]. Eco-plasticizers may also contribute to stability by decreasing diffusion of low-molecular-weight decomposition products [17].

The stability of propellants is a key parameter determining their long-term storage safety [18,19]. To standardize stability assessment procedures, NATO has developed a series of STANAG and AOP standards that define the conditions for accelerated aging and the corresponding evaluation criteria. The STANAG 4582 standard [20] specifies that propellants must remain stable for at least 10 years at 25 °C, whereas the AOP-48 [21] procedure establishes the allowable stabilizer depletion (a maximum of 80%) and a minimum residual stabilizer content of 0.2% after aging.

The chemical stability of nitrocellulose-based propellants is commonly assessed using analytical and thermal techniques such as high-performance liquid chromatography (HPLC) [22,23,24], gas chromatography [24,25], heat flow calorimetry (HFC) [26,27], differential scanning calorimetry, and thermogravimetric analysis [28]. These techniques enable both the determination of stabilizer depletion and the monitoring of exothermic reactions during aging. Traditional stability tests, including the Bergmann–Junk test, Abel test, vacuum stability test, and indicator paper methods, are still used as preliminary assessments. Recently, an increasing amount of attention has been paid to replacing classical stabilizers such as diphenylamine with natural compounds like curcumin, which have beneficial effects on thermal stability and lower toxicity [28,29].

Despite ongoing advancements, there is still a lack of systematic comparative studies on the performance of citrate plasticizers in nitrocellulose propellants containing nitrocellulose of different origins and with different nitrogen contents, especially in combination with NATO-standardized stability assessments. Furthermore, limited information exists regarding the incorporation efficiency of ATEC and ATBC and their simultaneous influence on the thermal behavior and energetic properties of single-base propellants. Therefore, the aim of this study was to evaluate the thermal and chemical stability of nitrocellulose propellants modified with triethyl 2-acetylcitrate (ATEC) and tributyl 2-acetylcitrate (ATBC) as well as assess their influence on density, the heat of combustion, and incorporation efficiency. Stability tests were performed in accordance with NATO STANAG 4582 [20] and AOP-48 standards [21] to verify the feasibility of replacing phthalates with citrate esters in propellant formulations.

## 2. Materials and Methods

### 2.1. Materials

Single-base propellants with a single perforation stabilized with centralite 1 (N,N′-Diethyl-N,N′-diphenylurea, CI) and with a layer thickness of 0.40 ± 0.05 mm were used for the modification processes. The propellants were manufactured by MESKO S.A. (Pionki, Poland). The investigated propellants, the type of nitrocellulose, and nitrogen content are summarized in Table 1.

The following reagents were used in the modification process:

- Triethyl 2-acetylcitrate (ATEC) (Sigma Aldrich, Poznań, Poland);
- Tributyl 2-acetylcitrate (ATBC) (Sigma Aldrich, Poznań, Poland);
- Ethanol with 98% purity (Hurtownia Odczynników Chemicznych Butra, Skierniewice, Poland);
- 2-Nitrodiphenylamine (2-NDPA), 99.8% purity, synthesized at the Department of High-Energetic Materials, Warsaw University of Technology.

For the chromatographic analysis of the propellants, the following reagents were employed:

- Acetonitrile (ACN) (Sigma Aldrich, Poznań, Poland);
- Methanol (MeOH) (Sigma Aldrich, Poznań, Poland);
- Anhydrous calcium chloride (CaCl_2_) (Avantor Performance Materials Poland S.A. Zabrze, Poland).

### 2.2. Modification Process

The modification process is based on the diffusive incorporation of the modifier into the combustion layer of nitrocellulose-based propellants. [30,31]. The process of modifying the propellants was carried out in a 1000 mL flat-bottom glass reactor equipped with a mechanical stirrer with adjustable speed, a thermocouple, and a distillation setup connected to a thermostat and a vacuum pump.

In each experiment, 200 g of propellant was placed in the reactor, followed by 250 mL of distilled water and 250 mL of ethanol. The mixture was stirred and heated to 70 °C, after which a modifier solution was added. This solution consisted of 1 phr of plasticizer (ATEC or ATBC) and 0.1 phr of 2-nitrodiphenylamine dissolved in a 5% ethanol solution. 2-NDPA was used as an indicator of the depth of penetration of the modifier. The mixture was conditioned for 2 h. After 1.5 h, an additional 250 mL of distilled water was added, and the thermostat temperature was raised to 75 °C.

In the next stage, vacuum distillation was applied for 2.5 h to remove ethanol. Subsequently, the temperature was lowered to 20 °C, and the propellant–water mixture was poured onto a metal sieve and dried at room temperature.

The dried propellant was then subjected to graphitization in a polishing mill containing polymer balls. Approximately 0.2 phr of graphite was added, and the process was carried out for 4 h. Two modified samples were prepared for each type of base propellant. The summary of all modifications is presented in Table 2.

### 2.3. Research Methods

Heat of combustion (Q) was determined using the adiabatic bomb calorimeter IKA C2000 Basic. The measurements were performed for 2.0000 ± 0.0005 g of sample under reduced pressure (3–4 mbar). A standard double-base propellant (2.0000 ± 0.0005 g, Q = 4914 J g^−1^) and steel wire (15 cm; 2.69 J g^−1^) were used to ignite the sample. The calorimeter was calibrated using a standard double-base propellant (Q = 4914 J g^−1^). The result was the mean value and standard deviation of two measurements for individual samples.

Density (d) was determined using a helium pycnometer (Micrometrics AccuPyc 1330) at a temperature of 25.0  ±  0.5 °C. To perform the test, a measuring vessel with a volume of 3.5 cm^3^ was filled with the tested material to approximately 75% of its volume. The final result was the average of ten measurements for each propellant; additionally, the calculated standard deviation values are provided.

The thickness of the combustible layer of the modified propellants was determined using a Delta Optical Smart 5M PRO optical microscope. The grains were sectioned to determine the thickness of the combustible layer. The cut was made along the channel using a scalpel.

The chemical stability of nitrocellulose-based propellants was determined using a TAM III calorimeter (TA Instruments, Warsaw, Poland) according to STANAG 4582 [20]. The sample mass was 2.0 g, and it was placed in a 4.3 mL calorimeter glass vessel. The prepared vessels with studied materials were placed in a calorimeter and thermostated at 70, 80, and 90 °C for 34.8, 10.6, and 3.43 days. This time corresponds to isothermal storage of materials at a temperature of 25 °C for 10 years.

Additionally, to meet the requirements of AOP-48 [21], both the base and modified propellants were subjected to accelerated aging tests in a chamber oven at the same three temperatures (70, 80, and 90 °C). After aging, the propellants were analyzed via high-performance liquid chromatography (HPLC) to determine stabilizer depletion and plasticizer content.

Additionally, to meet the requirements of AOP-48, both the base and modified propellants were subjected to accelerated aging tests in a chamber oven at the same three temperatures (70, 80, and 90 °C). After aging, the propellants were analyzed using high-performance liquid chromatography (HPLC) to determine stabilizer depletion and plasticizer content.

Calibration curves were prepared for the stabilizer (Centralite I) and modifiers (ATEC and ATBC). First, a working solution was prepared. Using an automatic pipette, we added 200 μL of the given plasticizer to a 10 mL volumetric flask, and then the volume was made up to the mark using acetonitrile. Calibration solutions were prepared in 100 mL volumetric flasks by adding the appropriate amount of the working solution (20 μL, 50 μL, 100 μL, 150 μL, and 200 μL) and then sequentially adding 50 mL of acetonitrile, 10 mL of a 2% aqueous CaCl_2_ solution, and 30 mL of distilled water to each flask. After about one hour, the flasks were filled to the mark with water. The concentrations of the ATBC and ATEC standards in the calibration solutions are shown in Table 3.

**Figure 1:**
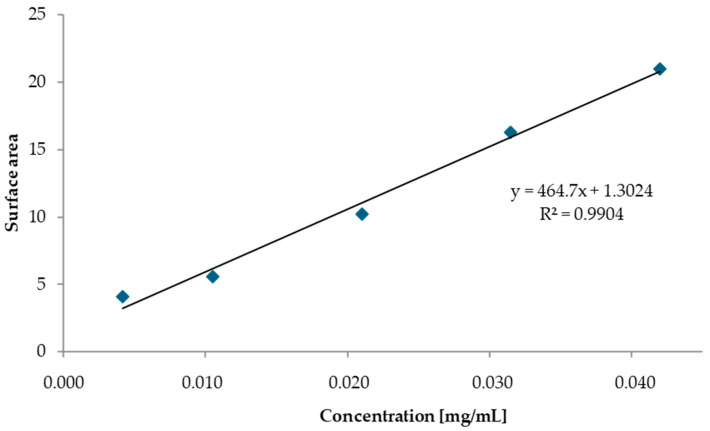
Calibration curve for ATBC obtained via HPLC.

**Figure 2:**
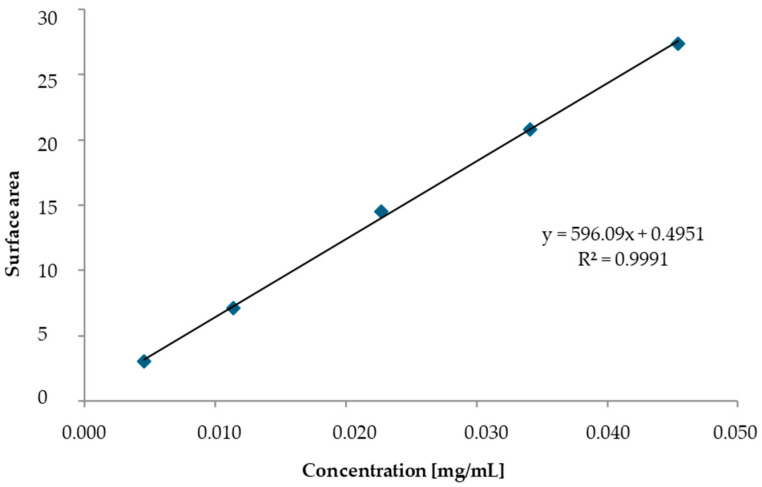
Calibration curve for ATEC obtained via HPLC.

Each calibration solution was injected three times, after which the peak areas were read from the chromatograms, and the mean peak area was determined. Calibration curves for ATEC and ATBC were plotted based on the mean peak area and the concentration of the standard substances (Figure 1 and Figure 2).

The quantitative determination was made according to the procedures described in AOP-48. The main analytical parameters of HPLC analysis are summarized in Table 4.

The analysis was carried out using an Agilent Technologies 1260 Infinity liquid chromatograph equipped with a guard column and a Supelcosil ABZ+PLUS column (150 mm × 4.6 mm, particle size of 5 µm). Table 4 presents the key parameters applied in the analytical procedure. Three injections were employed for each sample. Based on the peak areas obtained from the chromatograms and the previously established calibration curves, the amounts of individual compounds were calculated.

## 3. Results and Discussion

In the first stage of the study, the modified propellants were characterized in terms of their physical and energetic properties. Table 5 presents the parameters determined for both base and modified propellants, depending on the type of base nitrocellulose and modifier used.

Based on the data in Table 5, the highest heat of combustion was obtained for the unmodified (base) propellants. Lower combustion heat values were assigned to all the modified powders. The introduction of citric acid esters (ATEC and ATBC) reduced the heat of combustion, a result that is consistent with their negative oxygen balance. The introduction of modifiers with a negative oxygen balance reduces the heat of combustion, while energy modifiers such as nitroglycerin increase this parameter [32]. Since ATBC has a more negative oxygen balance than ATEC, the propellants containing ATBC exhibited lower calorific values. From an energetic point of view, modification with ATEC (M2) is therefore more favorable.

The modification process fills the pores within the subsurface combustion layer; therefore, the modified propellants are expected to exhibit a lower helium density [33]. Indeed, for all three base propellants (P1–P3), a decrease in density was observed after modification. The addition of ATEC (M2) caused a more pronounced reduction in density compared to that for ATBC (M1) for propellant P1. For P2, no significant differences between the two modifiers were observed, while for P3, the trend was opposite to that of P1, suggesting that the effect of the modifier may depend on the type of nitrocellulose used.

2-nitrodiphenylamine was introduced into the modification process together with the plasticizer. 2-NDPA has an intense orange color. Introducing it together with the modifier allows the thickness of the modified layer to be determined. 2-NDPA also has nitrocellulose-stabilizing properties. The migration of plasticizers to the inner combustible layer was similar for all the modified powders. The thickness of the combustible layer was approximately 0.040 mm. No influence of the plasticizer or nitrocellulose on the obtained values was observed. An important parameter in the evaluation of propellant modification is the efficiency of modifier incorporation. Quantitative chromatographic analyses were carried out to determine the actual amount of plasticizer incorporated into the nitrocellulose matrix. The results are summarized in Table 6.

**Figure 3:**
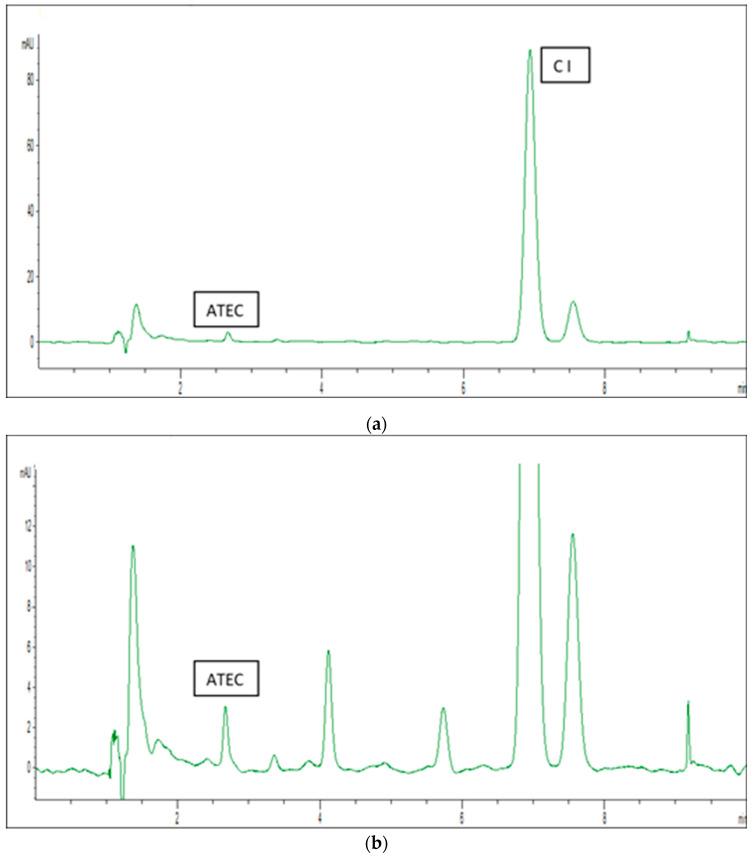
(**a**) Representative HPLC chromatogram of an ATEC-modified propellant. (**b**) The same chromatogram at a lower intensity scale.

**Figure 4:**
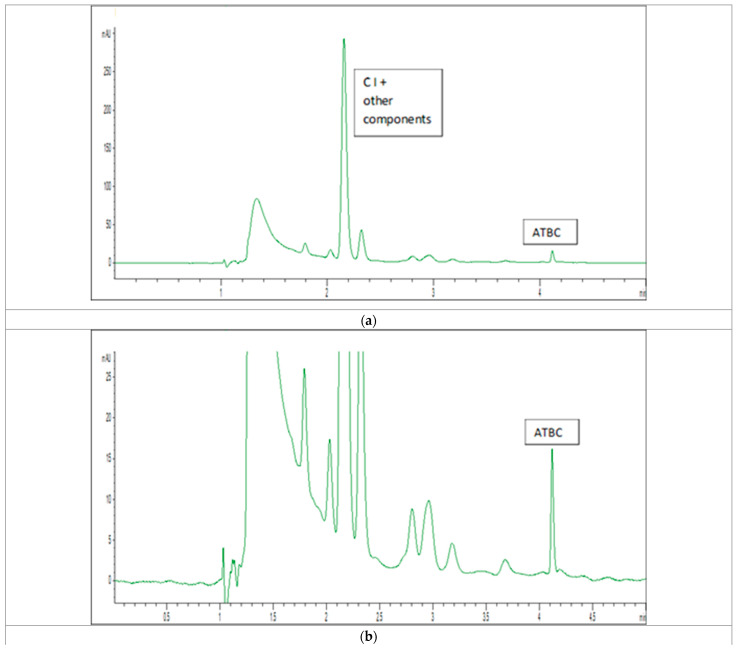
(**a**) Representative HPLC chromatogram of an ATBC-modified propellant. (**b**) The same chromatogram at a lower intensity scale.

For the propellants modified with ATBC and ATEC, the incorporation efficiency was over 100%. In the case of ATEC, the determined amount of modifier slightly exceeded the theoretical value introduced during processing. This can be attributed to peak tailing visible in the chromatograms or the coelution of small peaks (see Figure 3b and Figure 4b) with the plasticizer peak. This can lead to overestimation of peak area and, consequently, higher calculated analyte concentrations. As a reminder, the calibration curves were prepared for “pure” solutions, free from these impurities.

Figure 3 shows the chromatogram of the propellant modified with ATEC, including a zoomed-in version to allow better visualization of the peak shape.

The chromatogram for the ATBC-modified propellant is presented in Figure 4. The ATBC peak (retention time ≈ 4.1 min) was well-defined and symmetrical, without tailing or fronting, which allowed for more accurate quantification. However, it cannot be ruled out, as already discussed above for ATEC, that co-elution with impurities originating from the powders also occurred here.

### 3.1. Thermal Stability Assessment

The next stage of the investigation focused on the evaluation of propellant stability using two NATO-standardized methods: heat flow calorimetry (HFC) in accordance with STANAG 4582 and stabilizer depletion testing in accordance with AOP-48.

In the HFC method, samples were conditioned at 70, 80, and 90 °C using a TAM III microcalorimeter. The aging times and critical heat release values are summarized in Table 7.

**Figure 5:**
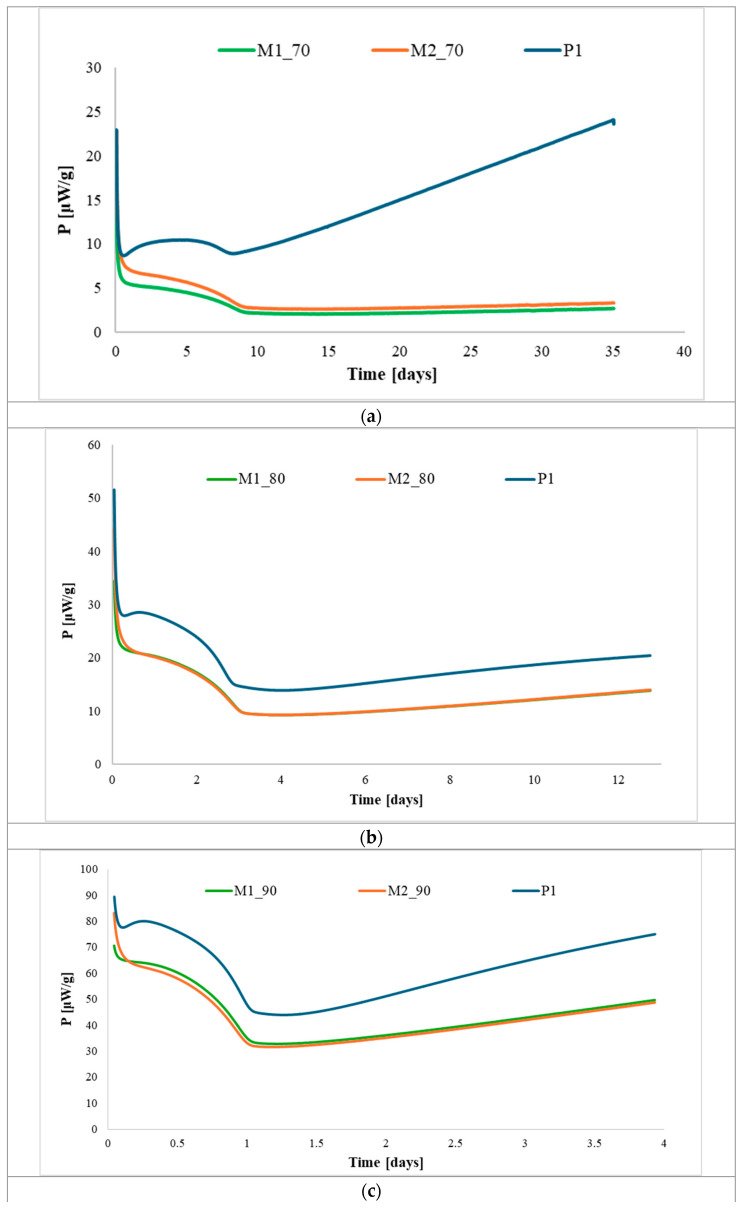
Heat-flow curves for propellants P1, M1, and M2 after accelerated aging at (**a**) 70 °C; (**b**) 80 °C; and (**c**) 90 °C.

**Figure 6:**
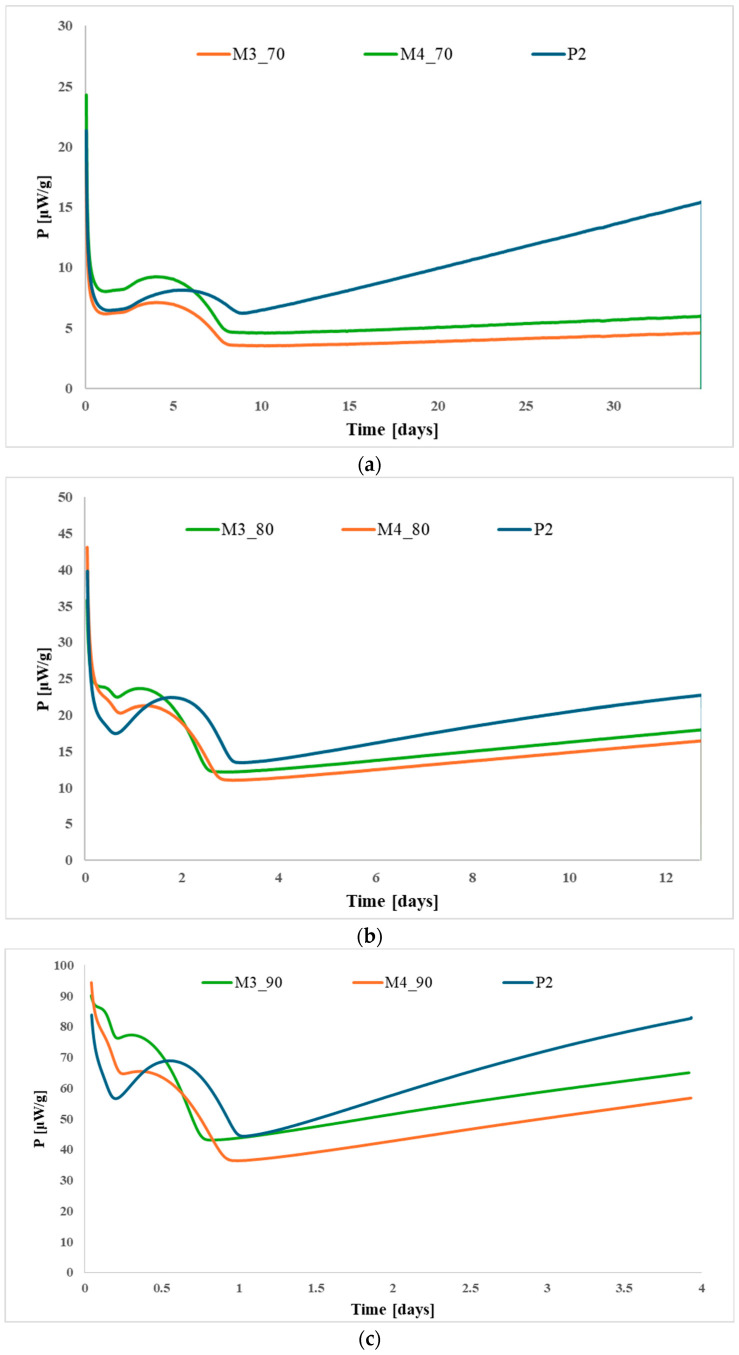
Heat-flow curves for propellants P2, M3, and M4 after accelerated aging at (**a**) 70 °C; (**b**) 80 °C; and (**c**) 90 °C.

**Figure 7:**
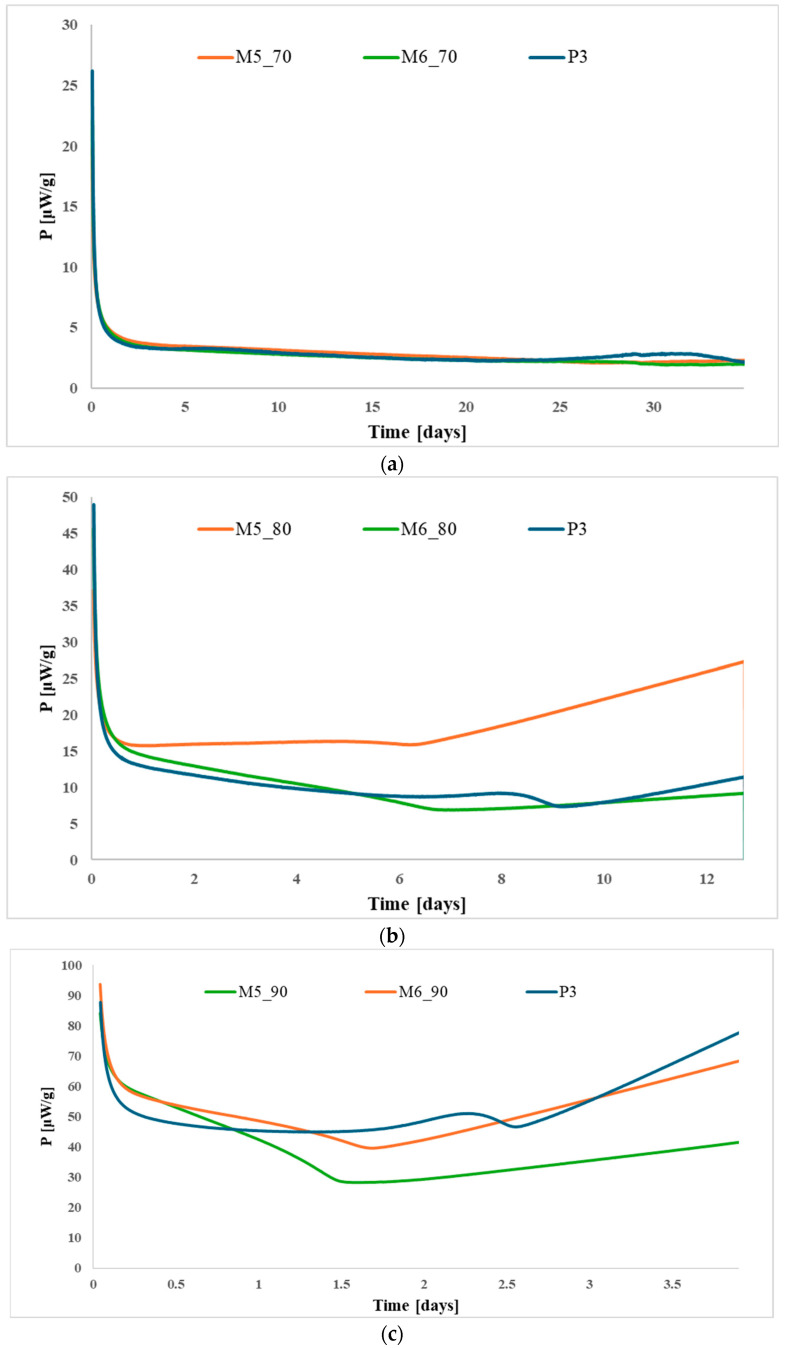
Heat-flow curves for propellants P3, M5, and M6 after accelerated aging at (**a**) 70 °C; (**b**) 80 °C; and (**c**) 90 °C.

Figure 5, Figure 6 and Figure 7 show the HFC curves for the modified propellants based on each type of nitrocellulose (P1–P3) at the studied temperatures.

Analysis of the HFC data shows that the aging behavior is comparable across all temperatures, indicating that temperature has only a minor effect on the rate at which the propellants degrade. None of the samples exceeded the maximum permissible heat flow, confirming that all the propellants studied are stable for at least 10 years of storage at 25 °C. Furthermore, the modified propellants exhibited lower heat release compared to the base propellants, suggesting that the incorporation of citric acid esters improved their thermal stability.

**Figure 8:**
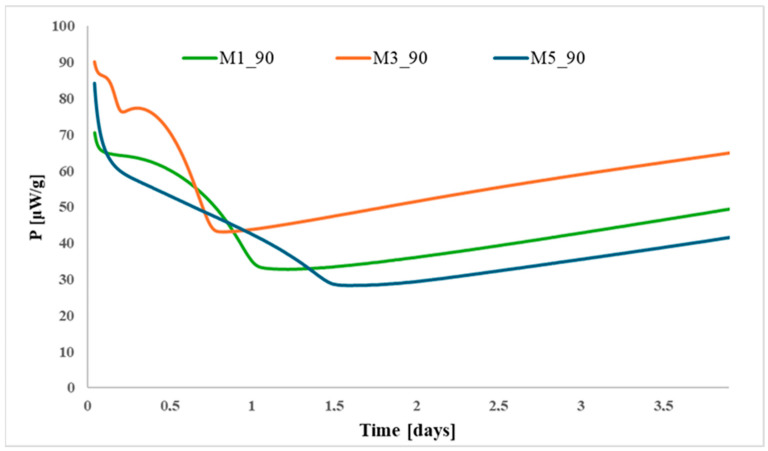
Comparison of HFC curves for propellants modified with ATBC prepared from different types of nitrocellulose (M1—wood base, 13.01% N; M3—wood base, 13.26% N; and M5—cotton base, 13.26% N).

Figure 8 illustrates the HFC curves for ATBC-modified propellants prepared with different types of nitrocellulose at 90 °C. Variations in the curve profiles indicate that the origin and nitrogen content of the nitrocellulose affect the thermal decomposition behavior and overall stability of the propellants.

### 3.2. Stabilizer Depletion Analysis

In accordance with AOP-48, stabilizer depletion was monitored chromatographically after the accelerated aging tests. The concentration of Centralite I (CI) was determined before and after conditioning at 70, 80, and 90 °C. The results are summarized in Table 8.

The data indicate that the stabilizer content decreased in all the propellants after aging, with the most significant loss observed for the base propellant P1, which was obtained from nitrocellulose with the lowest nitrogen content. The propellants based on nitrocellulose with a higher nitrogen content (P2) and cotton-based nitrocellulose (P3) showed better stability.

In contrast, for all the modified propellants, the stabilizer content remained nearly constant across the tested temperatures. The extent of stabilizer depletion never exceeded 80% of the initial content, and the residual amount of stabilizer was always above 0.2% by weight, fulfilling the NATO AOP-48 criteria. This confirms that the modified propellants will remain stable for at least ten years of storage at 25 °C. Moreover, citric acid esters did not negatively affect the chemical stability of the propellants.

## 4. Conclusions

The study provides the first detailed analysis of the effect of ATBC and ATEC citrate plasticizers on the stability of nitrocellulose powders, covering energy parameters, microstructure, and the behavior of stabilizers during aging.

The thermal and chemical stability of nitrocellulose-based propellants modified with triethyl 2-acetylcitrate (ATEC) and tributyl 2-acetylcitrate (ATBC) were evaluated using nitrocellulose with different origins and nitrogen contents. The incorporation of citric acid esters resulted in a reduction in the propellants’ heat of combustion, which can be attributed to their negative oxygen balance; simultaneously, they maintained favorable energetic and structural properties. Among the tested formulations, the propellants containing ATEC exhibited slightly higher calorific values compared to those modified with ATBC, likely due to subtle differences in the oxygen balance and ester–nitrocellulose interactions. Although this study provides data on the thickness of the modified layer, indicating the depth to which the plasticizer was successfully incorporated, further investigation is needed to assess plasticizer migration over time and under different conditions.

Accelerated aging studies, conducted in accordance with NATO STANAG 4582 [20] and AOP-48 standards [21], confirmed that all the modified propellants will remain chemically and thermally stable for at least ten years of storage at 25 °C. The addition of citric acid esters led to a decrease in the heat released during aging, indicating improved thermal stability. This suggests that citrates may also slow autocatalytic decomposition pathways in nitrocellulose. At the same time, stabilizer depletion remained within the acceptable limits defined by NATO standards, confirming the compatibility of the additives with the nitrocellulose matrix.

The obtained results indicate that citric acid derivatives such as ATEC and ATBC can serve as environmentally friendly and effective plasticizers for nitrocellulose propellants. Their use makes it possible to reduce reliance on toxic phthalates without compromising the performance, stability, or safety of the propellants. The tests were conducted on a laboratory series of propellants and within a limited temperature range, so they may not fully reflect storage conditions in military systems. In addition, ATEC chromatographic analysis revealed limitations of the method resulting from peak tailing, which may affect the accuracy of the determinations. Future research should focus on optimizing the proportions of eco-friendly plasticizers to achieve the best balance between energy efficiency and environmental safety. Future work will also include testing stability under other conditions, determining the extent of plasticizer migration, and improving the method for identifying citrates in a nitrocellulose matrix.

## Figures and Tables

**Table 1:** Characteristics of base propellants.

Base Propellant	Type of Nitrocellulose	Nitrogen Content [%]
P1	wood base	13.01
P2	wood base	13.26
P3	cotton-based (linter)	13.26

**Table 2:** Propellant modifications made.

Code	Base Propellant	Type of Modifier
M1	P1	ATBC
M2	P1	ATEC
M3	P2	ATBC
M4	P2	ATEC
M5	P3	ATBC
M6	P3	ATEC

**Table 3:** Concentrations of modifiers (ATBC AND ATEC) in calibration solutions.

Solution	1	2	3	4	5
Concentration of ATBC [mg/mL]	0.004	0.011	0.021	0.032	0.042
Concentration of ATEC [mg/mL]	0.005	0.011	0.023	0.034	0.045

**Table 4:** Analytical parameters for the determination of CI, ATBC, and ATEC via HPLC.

Analyte	Column Temperature [°C]	Mobile-Phase Composition	Flow Rate [mL/min]	Injection Volume [μL]	Wavelength [nm]
CI	50	14% ACN, 40% MeOH 46% water	1.5	10	210
ATBC	65	30% ACN, 40% MeOH 30% water	1.5	25	222
ATEC	50	14% ACN, 40% MeOH 46% water	1.5	25	222

**Table 5:** Physicochemical parameters of base and modified propellants.

Propellant	Modifier	Heat of Combustion [J/g]	Density [g/cm^3^]	The Thickness of the Combustible Layer [mm]
P1	-	4163 ± 1	1.659 ± 0.001	-
M1	ATBC	3841 ± 11	1.645 ± 0.001	0.035 ± 0.006
M2	ATEC	3933 ± 8	1.632 ± 0.001	0.036 ± 0.008
P2	-	4150 ± 7	1.656 ± 0.001	-
M3	ATBC	3902 ± 11	1.630 ± 0.001	0.040 ± 0.011
M4	ATEC	3949 ± 1	1.627 ± 0.001	0.039 ± 0.005
P3	-	4122 ± 2	1.644 ± 0.001	-
M5	ATBC	3821 ± 2	1.625 ± 0.001	0.038 ± 0.004
M6	ATEC	3860 ± 6	1.631 ± 0.001	0.041 ± 0.005

**Table 6:** Determined amounts of modifiers in propellants (target input: 1 phr).

Propellant	Modifier	Determined Amount [mg per 100 mg of Propellant]
M1	ATBC	1.00 ± 0.05
M2	ATEC	1.10 ± 0.05
M3	ATBC	0.96 ± 0.05
M4	ATEC	1.17 ± 0.05
M5	ATBC	1.01 ± 0.05
M6	ATEC	1.14 ± 0.05

**Table 7:** Aging times and maximum permissible heat flow values (STANAG 4582).

**Temperature [°C]**	**Aging Time [Days]**	**Maximum Heat Flow [μW/g]**
70	34.8	34.5
80	10.6	114
90	3.43	350

**Table 8:** Concentration of stabilizer (Centralite I) before and after artificial aging.

**Propellant**	**CI Content [mg per 100 mg of Propellant]**			
	**Before Aging**	**After Aging T = 90 °C**	**After Aging T = 80 °C**	**After Aging T = 70 °C**
P1	1.16 ± 0.05	0.66 ± 0.05	0.60 ± 0.05	0.78 ± 0.05
M1	1.09 ± 0.05	0.98 ± 0.05	0.90 ± 0.05	0.94 ± 0.05
M2	1.11 ± 0.05	0.96 ± 0.05	0.91 ± 0.05	0.92 ± 0.05
P2	1.11 ± 0.05	0.87 ± 0.05	0.87 ± 0.05	0.89 ± 0.05
M3	1.10 ± 0.05	0.98 ± 0.05	0.96 ± 0.05	0.96 ± 0.05
M4	1.10 ± 0.05	1.00 ± 0.05	0.96 ± 0.05	0.98 ± 0.05
P3	1.31 ± 0.05	1.24 ± 0.05	1.25 ± 0.05	1.20 ± 0.05
M5	1.29 ± 0.05	1.22 ± 0.05	1.20 ± 0.05	1.22 ± 0.05
M6	1.29 ± 0.05	1.22 ± 0.05	1.22 ± 0.05	1.23 ± 0.05

## Data Availability

The data presented in this study are available on request from the corresponding author. The data are not publicly available due to institutional restrictions.

## Abbreviations

The following abbreviations are used in this manuscript:

DBP	Dibutyl phthalate
ATEC	Triethyl 2-acetylcitrate
ATBC	Tributyl 2-acetylcitrate
CI	N,N′-Diethyl-N,N′-diphenylurea
HPLC	High-performance liquid chromatography
HFC	Heat flow microcalorimetry

## References

[1] Anastas P.T., Warner J.C. (1998). Principles of Green Chemistry. Green Chemistry: Theory and Practice.

[2] Regulation (EC) No 1907/2006 of The European Parliament and of the Council of 18 December 2006 the Registration, Evaluation, Authorisation and Restriction of Chemicals (REACH), Establishing a European Chemicals Agency, Amending Directive 1999/45/EC and Repealing Council Regulation (EEC) No 793/93 and Commission Regulation (EC) No 1488/94 as Well as Council Directive 76/769/EEC and Commission Directives 91/155/EEC, 93/67/EEC, 93/105/EC and 2000/21/EC 2006. https://eur-lex.europa.eu/eli/reg/2006/1907/oj.

[3] Chavez D.E. (2014). The Development of Environmentally Sustainable Manufacturing Technologies for Energetic Materials. Green Energetic Mater..

[4] Boulkadid M.K., Lefebvre M.H., Jeunieau L., Dejeaifve A. (2021). Assessment of the Migration of Combustion Moderator in Nitrocellulose-Based Propellant. Materials Horizons: From Nature to Nanomaterials.

[5] Gańczyk-Specjalska K., Cieślak K., Jakubczak M., Drożdżewska-Szymańska K., Tomaszewski W., Prasuła P. (2023). The Effect of Citrate Plasticizers on the Properties of Nitrocellulose Granules. Propellants Explos. Pyrotech..

[6] Cao X., Nan F., Fan W., Gao H., Chen L., Guo Y., He W. (2024). Preparation, Formation Mechanism, and Performance of Nitrocellulose Aqueous Coating Based on the Anti-Solvent Method. Colloids Surf. A Physicochem. Eng. Asp..

[7] Mendonça-Filho L.G., Rodrigues R.L.B., Rosato R., Galante E.B.F., Nichele J. (2019). Combined Evaluation of Nitrocellulose-Based Propellants: Toxicity, Performance, and Erosivity. J. Energetic Mater..

[8] Jia P., Xia H., Tang K., Zhou Y. (2018). Plasticizers Derived from Biomass Resources: A Short Review. Polymers.

[9] Muobom S.S., Umar A.-M.S., Brolin A.-P., Soongseok Y. (2020). A Review on Plasticizers and Eco-Friendly Bioplasticizers: Biomass Sources and Market. Int. J. Eng. Res. Technol..

[10] Krutko I., Yavir K., Kaulin V., Strankowski M. (2018). Effect of Antioxidants on the Stability of Pitch-Based Polymer to Thermo-Oxidative Action. Chem. Chem. Technol..

[11] Rusly S.N.A., Jamal S.H., Samsuri A., Mohd Noor S.A., Abdul Rahim K.S. (2024). Stabilizer Selection and Formulation Strategies for Enhanced Stability of Single Base Nitrocellulose Propellants: A Review. Energetic Mater. Front..

[12] Rusly S.N.A., Jamal S.H., Samsuri A., Mohd Noor S.A., Abdul Rahim K.S. (2024). A Green Stabilizer for Nitrate Ester-Based Propellants: An Overview. Heliyon.

[13] Chebbah M., Tarchoun A.F., Benaliouche F., Abdelaziz A., Trache D. (2025). Advancing Nitrocellulose Thermal Stability through the Incorporation of Ion-Exchanged ZSM-5 Zeolite for Enhanced Performance. FirePhysChem.

[14] Cieślak K., Gańczyk-Specjalska K., Drożdżewska-Szymańska K., Królikowska M., Jakubczak M. (2022). Physicochemical Properties and Thermal Behavior of Nitrocellulose Granules with Eutectic Mixtures of Stabilizers. J. Therm. Anal. Calorim..

[15] Qi X., Li H., Zhao Y., Yan N. (2019). Comparison of the Structural and Physical Properties of Nitrocellulose Plasticized by N-Butyl-N-(2-Nitroxy-Ethyl) Nitramine and Nitroglycerin: Computational Simulation and Experimental Studies. J. Hazard. Mater..

[16] Yang L., Wu X., Li J., Chen T., Liu M., He Q. (2021). Structure and Property of Propellant Based on Nitroglycerine/Glycerol Triacetate Mixed Plasticizers: Molecular Dynamics Simulation and Experimental Study. R. Soc. Open Sci..

[17] Agrawal J.P. (2010). High Energy Materials: Propellants, Explosives and Pyrotechnics.

[18] De Klerk W.P.C. (2015). Assessment of Stability of Propellants and Safe Lifetimes. Propellants Explos. Pyrotech..

[19] Cieślak K., Tomaszewski W., Gańczyk-Specjalska K. (2023). Study of the Effect of Accelerated Ageing on the Properties of Modified Nitrocellulose Propellants. High Energy Mater..

[20] (2007). Explosives, Nitrocellulose Based Propellants, Stability Test Procedure And Requirments Using Heat Flow Calorimetry.

[21] (2008). Explosives, Nitrocellulose-Based Propellants, Stability Test Procedures And Requirements Using Stabilizer Depletion.

[22] Lindblom T. (2002). Reactions in Stabilizer and Between Stabilizer and Nitrocellulose in Propellants. Propellants Explos. Pyrotech..

[23] Boers M.N., De Klerk W.P.C. (2005). Lifetime Prediction of EC, DPA, Akardite II and MNA Stabilized Triple Base Propellants, Comparison of Heat Generation Rate and Stabilizer Consumption. Propellants Explos. Pyrotech..

[24] Chajistamatiou A.S., Bakeas E.B. (2016). A Rapid Method for the Identification of Nitrocellulose in High Explosives and Smokeless Powders Using GC–EI–MS. Talanta.

[25] Mazur I., Kasprzak P., Borkowski J. (2025). Safety Monitoring of Storage and Use of Solid Homogeneous Rocket Propellants Through the Chemical Composition Analysis. Def. Sci. J..

[26] Wilker S., Heeb G., Vogelsanger B., Petržílek J., Skládal J. (2007). Triphenylamine—A “new” Stabilizer for Nitrocellulose Based Propellants—Part I: Chemical Stability Studies. Propellants Explos. Pyrotech..

[27] Fryš O., Bajerová P., Eisner A., Skládal J., Ventura K. (2011). Utilization of New Non-Toxic Substances as Stabilizers for Nitrocellulose-Based Propellants. Propellants Explos. Pyrotech..

[28] Rodrigues R.L.B., Gomes Buitrago P.A., Nakano N.L., Peixoto F.C., Lemos M.F., França T.C.C., Mendonça Filho L.G. (2022). Can Green Nitrocellulose-Based Propellants Be Made through the Replacement of Diphenylamine by the Natural Product Curcumin?. J. Energetic Mater..

[29] Gańczyk-Specjalska K. (2019). Conventional and Alternative Nitrocellulose Stabilisers Used in Gun Propellants. High Energy Mater..

[30] Jones A., Warrender G., Porter D., Barber N. A Reduced Toxicity Deterrent for Single Base Propellants. Proceedings of the IMEMTS 2015.

[31] Cieślak K., Gołofit T., Tomaszewski W., Chmielarek M., Maksimowski P., Pawłowski W. (2021). Modification of the Burning Layer of Nitrocellulose Powders with Liquid Nitroesters. High Energy Mater..

[32] Liu B., Ma F.S., Bian X.Y., Tian D.Q., Wang Q.L., Lv H. (2023). Research on the Performance of Deterred-Coating DIANP Gun Propellant. Journal of Physics: Conference Series.

[33] Cieślak K., Gańczyk-Specjalska K. (2022). Methods of Modifying Single Base Propellants Using Centralite I, Dibutyl Phthalate and Rosin. High Energy Mater..

